# Motor Scooter Syndrome Revisited: A Case of Delayed Presentation of Traumatic Occlusion of the Common Femoral Artery

**DOI:** 10.7759/cureus.43150

**Published:** 2023-08-08

**Authors:** Joseph Boscia, Thomas Sanders, Saptarshi Biswas

**Affiliations:** 1 Surgery, University of South Carolina School of Medicine, Columbia, USA; 2 Surgery, Allegheny Health Network, Pittsburgh, USA

**Keywords:** rhabdomyolysis, arterial reconstruction, blunt vascular trauma, motor scooter syndrome, common femoral artery occlusion

## Abstract

Injuries to the common femoral artery (CFA) are usually associated with local fractures. Other common mechanisms of injury include intimal disruption, intramural hematomas, and subintimal fibrosis. Occlusions to the CFA may also result from blood clots or arterial emboli via blunt injury. Blunt trauma causing injury to the common femoral artery is exceedingly rare. Blunt injury to the CFA may be caused by “motor-scooter-handlebar syndrome.” We present a unique case where the delayed diagnosis of such an injury led to acute renal failure, rhabdomyolysis, and prolonged morbidity.

## Introduction

Blunt trauma causing injury to the common femoral artery (CFA) is rare. When present, these injuries usually occur in association with fractures [1,2]. Intimal disruption, intramural hematomas, and subintimal fibrosis are other common injury mechanisms. Subsequent occlusions may result from associated thromboses. Vascular injuries result from blunt trauma in 5% to 25% of vascular injuries [3]. An uncommon cause of blunt CFA injury is motorcycle or bicycle handlebar trauma, wherein the end of the handlebar causes blunt injury [4-6]. Compression to the inguinal ligament is postulated as the causative mechanism. The initial diagnosis is difficult because of how infrequently this injury mechanism occurs. Unfortunately, a delay in diagnosing this condition can lead to further disease progression.

One such progression is acute kidney injury. Trauma patients are generally susceptible to acute kidney injury via rhabdomyolysis, hyperperfusion of fluids secondary to hemorrhagic shock, and other factors [7]. Acute kidney injury is subsequently associated with higher morbidity and mortality [8]. This case report presents a delayed diagnosis of CFA occlusion caused by motorcycle handlebar trauma leading to acute renal failure and rhabdomyolysis.

This article was previously presented as a meeting abstract at the 2018 Royal Australasian College of Surgeons May 7th, 2018. This article will be presented as a meeting abstract at the 2023 Southeastern Surgical Conference February 14th, 2023.

## Case presentation

A 43-year-old male presented to the Emergency Department (ED) as a Level 2 trauma with the chief complaint of right-sided groin and shoulder pain following a motorcycle accident. He was unhelmeted and appeared intoxicated on arrival. The patient was alert and oriented on arrival. However, he was unconscious on the scene, per Emergency Medical Services. He denied headaches, neck pain, back pain, chest pain, difficulty breathing, or abdominal pain. Vital signs recorded showed a temperature of 36.4°C, heart rate of 94, blood pressure of 163/94, respiratory rate of 18, and O_2_ saturation of 97%.

On physical exam, upper and lower extremity strength was 5/5 bilaterally. Dorsalis pedis and posterior tibial pulses were 2+ in all four extremities. The skin was warm and dry. He had significant ecchymoses to the right shoulder and right groin. The patient appeared agitated and slightly confused. The rest of the system review showed no gross abnormality. The patient had no significant past medical or surgical history.

A trauma computed tomography (CT) scan, including a head and neck non-contrast CT, a chest, abdomen, and pelvis contrast CT, and a CT Angiogram (CTA) of the neck, was performed. Imaging revealed a distal left vertebral artery dissection without severe stenosis and a non-displaced posterior right first rib fracture. 

**Figure 1:**
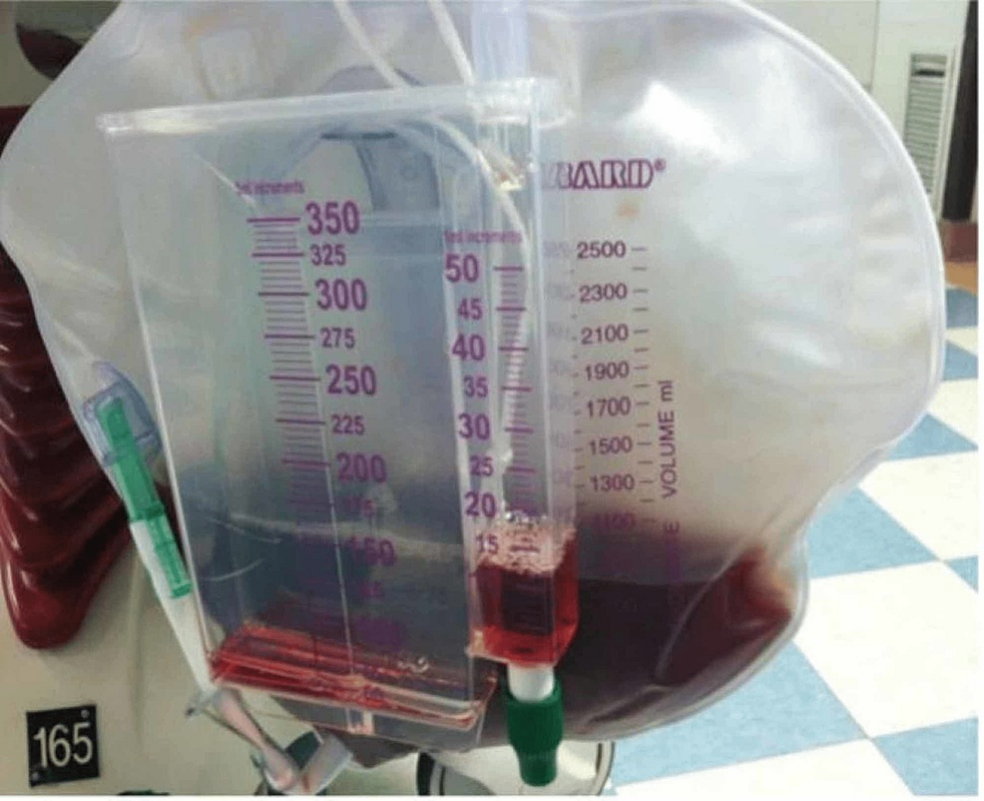
Tea-colored urine suggestive of rhabdomyolysis.

On returning from the CT scanner, the patient became increasingly agitated and was placed in 4-point restraints. He was initially admitted to the step-down unit. The following morning, he went into respiratory distress and had to be intubated and sedated. Cardiology was consulted for a troponin elevation (0.11 ng/mL). Creatinine phosphokinase (CK) was 19,270 and white blood cell count was elevated at 23.07 x 10^3^. Tea-colored urine with 2+ protein and 3+ blood was observed (Figure 1). Urine specific gravity was 1.036. A diagnosis of rhabdomyolysis was made.

Overnight, nursing noticed the right foot to be cold and pulseless. An arterial Doppler ultrasound was ordered, which showed a right common femoral artery occlusion with limited blood flow. The right foot was cold and mottled, with a dry eschar over the medial aspect of the mid-calf. They also noted rigor involving the right calf muscles, making the ankle and foot unable to plantar or dorsiflex. His contralateral left foot was warm with a palpable dorsalis pedis pulse.

**Figure 2:**
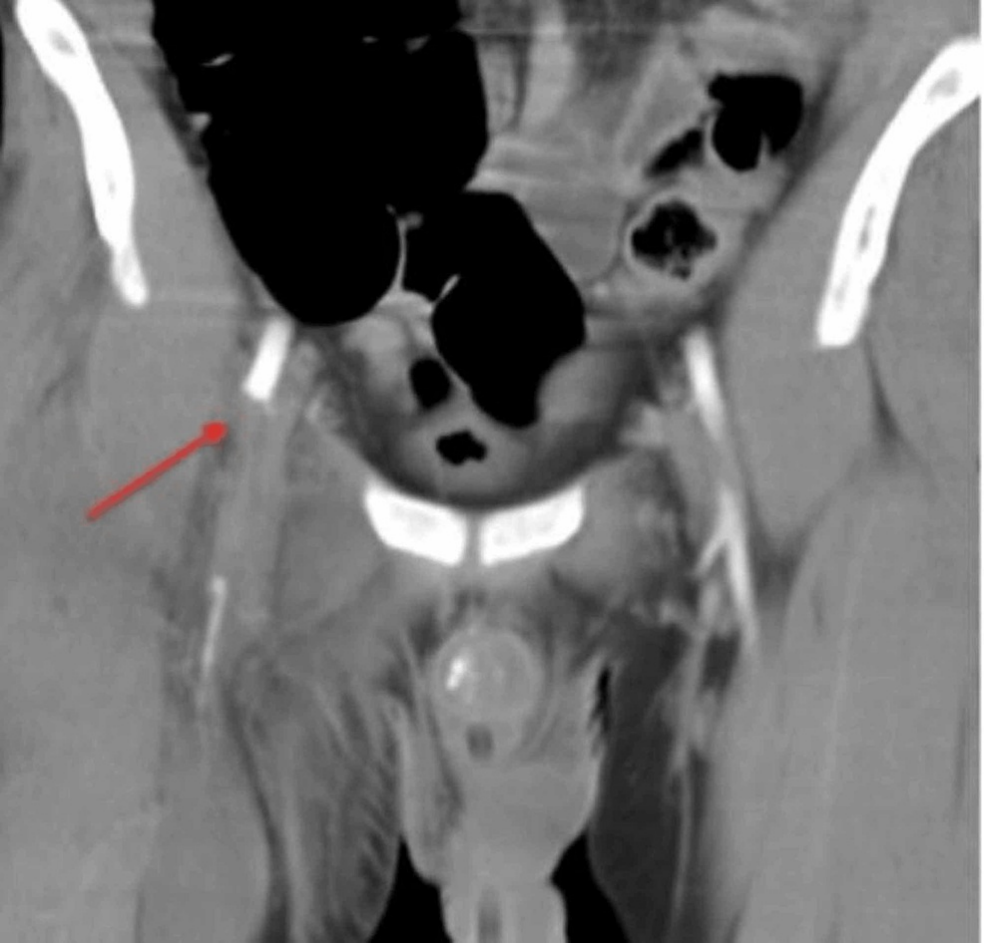
Computed Tomography Angiogram (CTA) showing 5 cm occlusion of the right common femoral artery (CFA) (red arrow).

**Figure 3:**
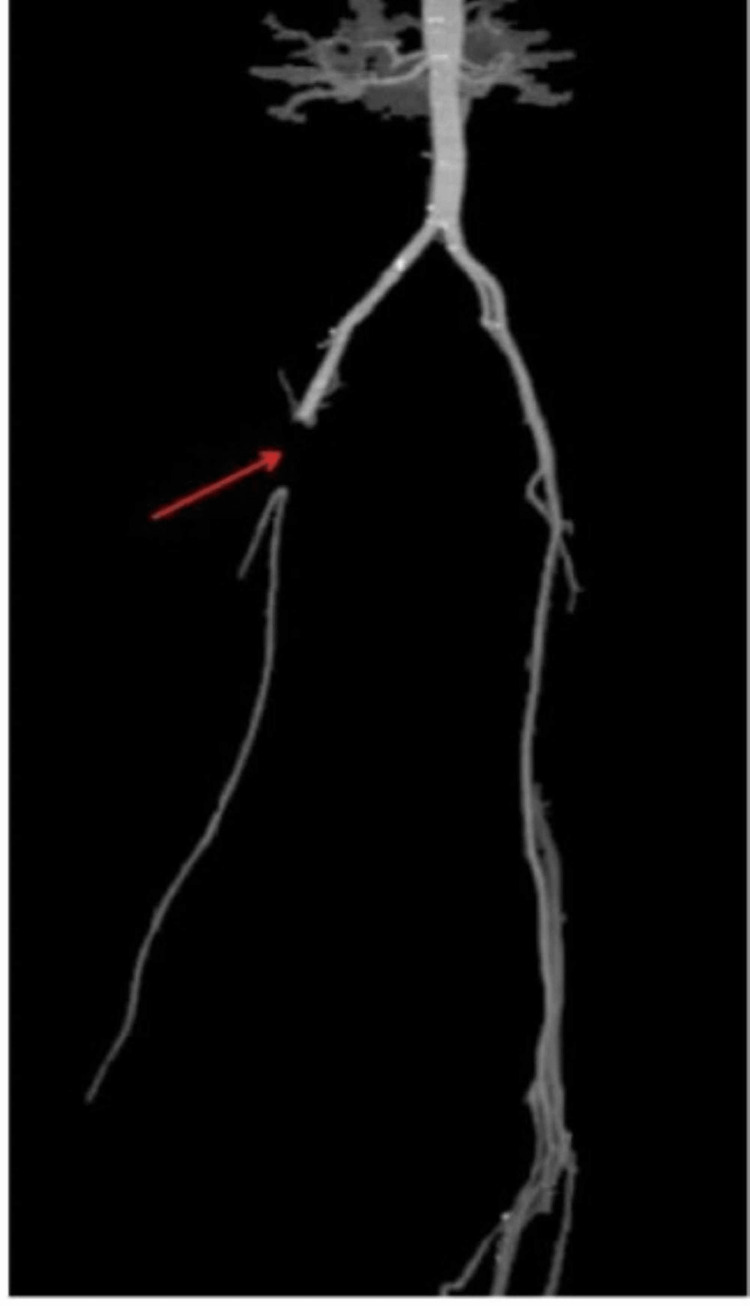
CT Angiogram (CTA) showing abrupt disruption of the common femoral artery (CFA) (red arrow).

CTA showed a 5 cm occlusion of the right CFA with reconstitution within the superficial femoral artery, leading to ischemia of the right lower extremity (Figure 2, 3).

**Figure 4:**
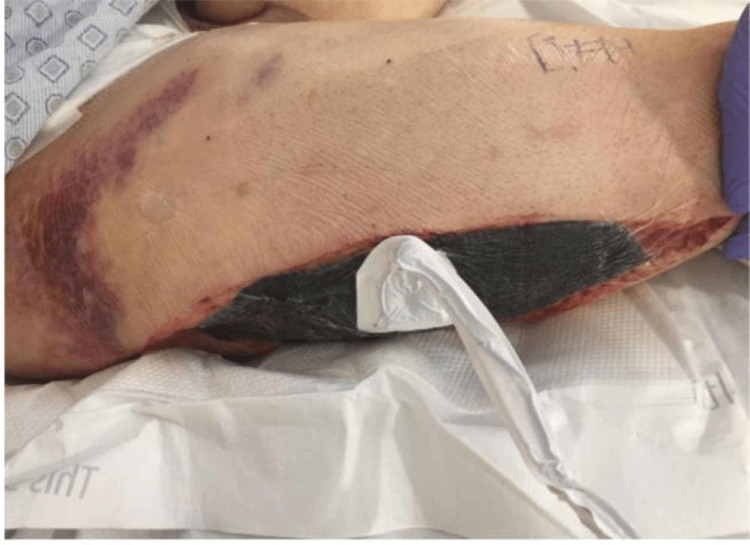
Inguinal hematoma and thigh fasciotomy.

**Figure 5:**
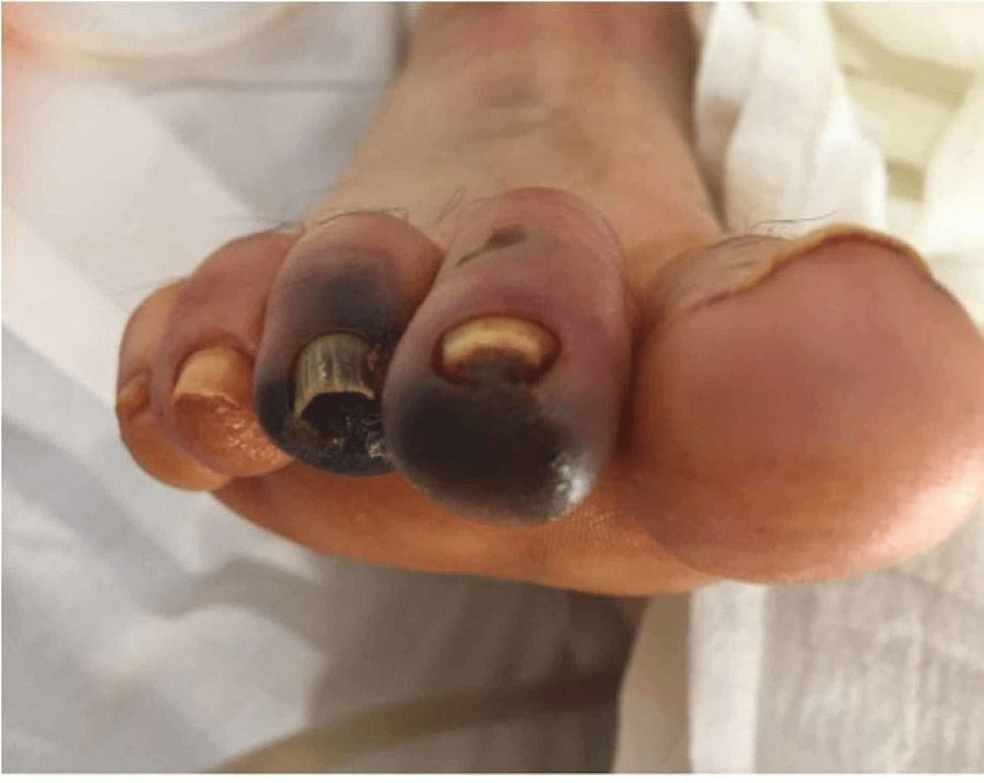
Ischemic changes to the foot.

The patient went to the operating room for a right femoral artery bypass. A semioblique incision was made along the right groin, and the right common femoral artery was dissected out. No pulse was found. However, the external iliac was found to have a strong pulse. A hematoma that had thrombosed was found in the entire wall of the femoral artery. The tunica media and tunica intima were disrupted at the arterial injury site. A longitudinal arteriotomy extended down to the femoral bifurcation and showed patent profunda and superficial femoral arteries. An 8 mm Dacron interposition graft was used to revascularize. The flow was then reestablished, resulting in strong pulses with multiphasic signals. Fasciotomies were performed to include the anterior and lateral, as well as the deep and superficial posterior compartments. The posterior tibial pulses were also palpable. However, the next day, his right pedal pulse was again absent, and he developed a tense right thigh leading to thigh fasciotomies and ischemic changes to the foot (Figure 4, 5). He continued on aggressive fluid resuscitation and supportive intensive care unit (ICU) management. Nephrology and infectious disease (ID) were on board. The patient remained extremely agitated off sedation, and on day 10, a tracheostomy and a percutaneous endoscopic gastrostomy (PEG) tube were performed. The patient was eventually stabilized and discharged. At a one-week follow-up appointment, the patient's right lower extremity demonstrated improvement and did not require further workup at that time. The patient was then lost to follow-up.

## Discussion

At the inguinal ligament, the femoral artery is superficial as it passes over the superior pubic ramus and the femoral head. Tethering from arterial branches, periadventitious connective tissue, and the femoral sheath makes the femoral artery relatively immobile, thus making it vulnerable to compression against the underlying bony structures [9].

Cases of injury to arteries of the lower extremities have been well reported in the literature [1,6,10-13]. But, blunt injury to the common femoral artery not associated with a fracture is less common. In this mechanism of injury, the CFA suffers direct trauma from the motorcycle or bicycle handlebar as the rider falls forward. The front wheel and handlebars of the motorcycle scooter or bicycle rotate to a perpendicular plane to the rider during a fall so that the point of impact is on the handlebar end [14]. This mechanism multiplies and concentrates the impact force over a relatively narrow area [15].

Maintaining a high degree of suspicion is the key to preventing delay in diagnosing injuries where the forces experienced may appear insignificant at first. Findings of bruits, pulse deficits, expanding hematomas, arterial bleeding, poor capillary filling, and cold extremities may narrow the differential. Vascular damage may not manifest initially. But, later findings of thrombosis, subintimal hemorrhage, dissection, or aneurysmal dilatation, often resulting in hemorrhage, pain, and ischemic changes at a remote site later on, may indicate this initial insult [16].

Color Doppler ultrasound, arteriography, and CT angiography are helpful diagnostic tools to rule out arterial injury. Once diagnosed, early heparinization followed by surgical repair, if indicated, is necessary [1]. Primary repair is advocated whenever possible. The extent of damage can often make primary repair difficult, especially in blunt trauma. Autogenous conduits, like the great saphenous veins, are usually preferred when grafting is necessary. Endovascular interventions are also reported for treating traumatic arterial injuries, although mostly in penetrating trauma [17]. When arterial reconstructions are required in children, interrupted sutures are used with wide spatulation of the anastomosis. If bypass grafts are used, they are configured to preserve vital collateral pathways [9].

Fasciotomy is an important adjunct in extremity arterial injuries. Although a clinical diagnosis, some authors recommend it routinely when the limb ischemia time exceeds six hours. However, absolute ischemia time is never the only determining factor of the degree of ischemia in the limb [16]. 

## Conclusions

The diagnosis of occlusion to the CFA must remain on the differential for any patients presenting with trauma to that region. The key to diagnosis is clinical suspicion and thus including CFA injury in the differential when appropriate is essential. In any traumatic injury presentation, the presence of red flag symptoms such as bruits, pulse deficits, expanding hematomas, arterial bleeding, poor capillary filling, and cold extremities may aid diagnosis. The presentation and discussion above aim to guide clinicians in the future diagnosis and successful treatment of this disease process.

## References

[1] Cargile JS 3rd, Hunt JL, Purdue GF (1992). Acute trauma of the femoral artery and vein. J Trauma.

[2] Asmar S, Bible L, Chehab M (2021). Traumatic femoral artery injuries and predictors of compartment syndrome: a nationwide analysis. J Surg Res.

[3] Feliciano DV, Moore FA, Moore EE (2011). Evaluation and management of peripheral vascular injury. Part 1. Western Trauma Association/critical decisions in trauma. J Trauma.

[4] Rozycki GS, Tremblay LN, Feliciano DV, McClelland WB (2003). Blunt vascular trauma in the extremity: diagnosis, management, and outcome. J Trauma.

[5] Deutsch V, Sinkover A, Bank H (1968). The motor-scooter-handlebar syndrome. Lancet.

[6] Yoshimura K, Hamamoto H (2019). Traumatic right common femoral artery occlusion caused by blunt bicycle handlebar injury: a case report. Surg Case Rep.

[7] Harrois A, Libert N, Duranteau J (2017). Acute kidney injury in trauma patients. Curr Opin Crit Care.

[8] Perkins ZB, Captur G, Bird R, Gleeson L, Singer B, O'Brien B (2019). Trauma induced acute kidney injury. PLoS One.

[9] Sarfati MR, Galt SW, Treiman GS, Kraiss LW (2002). Common femoral artery injury secondary to bicycle handlebar trauma. J Vasc Surg.

[10] Rich NM, Hobson RW, Fedde CW, Collins GJ (1975). Acute common femoral arterial trauma. J Trauma.

[11] Stanton PE Jr, Brown R, Rosenthal D, Clark M, Lamis PA (1986). External iliac artery occlusion by bicycle handle injury. J Cardiovasc Surg (Torino).

[12] Kioumehr F, Yaghmai I, Bakody P (1989). Delayed common femoral artery stenosis due to blunt trauma. Can Assoc Radiol J.

[13] Roth JW, Boyd CR (1999). Recreational bicycling and injury to the external iliac artery. Am Surg.

[14] Winston FK, Shaw KN, Kreshak AA, Schwarz DF, Gallagher PR, Cnaan A (1998). Hidden spears: handlebars as injury hazards to children. Pediatrics.

[15] Clarnette TD, Beasley SW (1997). Handlebar injuries in children: patterns and prevention. Aust N Z J Surg.

[16] Hafez HM, Woolgar J, Robbs JV (2001). Lower extremity arterial injury: results of 550 cases and review of risk factors associated with limb loss. J Vasc Surg.

[17] Xu Y, Xu W, Wang A (2019). Diagnosis and treatment of traumatic vascular injury of limbs in military and emergency medicine: a systematic review. Medicine (Baltimore).

